# Standardized, Systemic Phenotypic Analysis of *Umod* ^*C93F*^ and *Umod* ^*A227T*^ Mutant Mice

**DOI:** 10.1371/journal.pone.0078337

**Published:** 2013-10-24

**Authors:** Elisabeth Kemter, Petra Prückl, Birgit Rathkolb, Kateryna Micklich, Thure Adler, Lore Becker, Johannes Beckers, Dirk H. Busch, Alexander A. Götz, Wolfgang Hans, Marion Horsch, Boris Ivandic, Martin Klingenspor, Thomas Klopstock, Jan Rozman, Anja Schrewe, Holger Schulz, Helmut Fuchs, Valérie Gailus-Durner, Martin Hrabé de Angelis, Eckhard Wolf, Bernhard Aigner

**Affiliations:** 1 Chair for Molecular Animal Breeding and Biotechnology, and Laboratory for Functional Genome Analysis (LAFUGA), Gene Center, LMU, Munich, Munich, Germany; 2 German Mouse Clinic, Institute of Experimental Genetics, Helmholtz Zentrum München, German Research Center for Environmental Health, Neuherberg, Germany; 3 Institute of Medical Microbiology, Immunology, and Hygiene, TU, Munich, Munich, Germany; 4 Department of Neurology, Friedrich-Baur-Institut, LMU, Munich, Munich, Germany; 5 Member of German Center for Diabetes Research (DZD), Neuherberg, Germany; 6 Chair of Experimental Genetics, Center of Life and Food Sciences Weihenstephan, TU, Munich, Freising-Weihenstephan, Germany; 7 German Mouse Clinic, Comprehensive Pneumology Center, Institute of Lung Biology and Disease, Helmholtz Zentrum München, German Research Center for Environmental Health, Neuherberg, Germany; 8 Department of Medicine III, Division of Cardiology, University of Heidelberg, Heidelberg, Germany; 9 Molecular Nutritional Medicine, Else Kröner-Fresenius Center, TU, Munich, Freising-Weihenstephan, Germany; 10 German Center for Vertigo and Balance Disorders, University Hospital Munich, Campus Grosshadern, Munich, Germany; 11 Institute of Epidemiology I, Helmholtz Zentrum München, Neuherberg, Germany; IGBMC/ICS, France

## Abstract

Uromodulin-associated kidney disease (UAKD) summarizes different clinical features of an autosomal dominant heritable disease syndrome in humans with a proven uromodulin (*UMOD*) mutation involved. It is often characterized by hyperuricemia, gout, alteration of urine concentrating ability, as well as a variable rate of disease progression inconstantly leading to renal failure and histological alterations of the kidneys. We recently established the two *Umod* mutant mouse lines *Umod* ^*C93F*^ and *Umod* ^*A227T*^ on the C3H inbred genetic background both showing kidney defects analogous to those found in human UAKD patients. In addition, disease symptoms were revealed that were not yet described in other published mouse models of UAKD. To examine if further organ systems and/or metabolic pathways are affected by *Umod* mutations as primary or secondary effects, we describe a standardized, systemic phenotypic analysis of the two mutant mouse lines *Umod* ^*A227T*^ and *Umod* ^*C93F*^ in the German Mouse Clinic. Different genotypes as well as different ages were tested. Beside the already published changes in body weight, body composition and bone metabolism, the influence of the *Umod* mutation on energy metabolism was confirmed. Hematological analysis revealed a moderate microcytic and erythropenic anemia in older *Umod* mutant mice. Data of the other analyses in 7-10 month-old mutant mice showed single small additional effects.

## Introduction

The uromodulin gene (*UMOD*) encodes the uromodulin glycoprotein which is the most abundant urinary protein in mammals. It is synthesized exclusively and abundantly in the cells of the thick ascending limb of Henle’s loop (TALH) without macula densa cells. Once synthesized, the uromodulin precursor is processed within the endoplasmic reticulum (ER) and Golgi complex into mature glycoprotein, transported to the luminal membrane and excreted with urine.

In humans, usually heterozygous *UMOD* mutations result in a dominant heritable disease syndrome which is summarized as uromodulin-associated kidney disease (UAKD) or uromodulin storage disease. The disease is characterized by TALH dysfunction due to disturbed mutant uromodulin trafficking that in consequence leads to reduced fractional excretion of uric acid, leading in most cases to gout, mild defects of urine concentrating ability, as well as a variable rate of disease progression inconstantly leading to renal failure. Histological alterations of the kidneys include tubulointerstitial fibrosis with moderate inflammatory cell infiltrates and cysts [1,2].

To date, various *Umod* mutant mouse models are published. Two different *Umod* functional knockout models were established on the genetic background of 129/Sv and/or C57BL/6 inbred mice [3,4]. Compared to wild-type mice, homozygous knockout mice of both lines showed normal development and growth as well as behavior and fertility. The histological kidney structures also were not different, and they exhibited only minor changes in steady-state renal function. This indicates that loss of uromodulin expression is not sufficient to cause UAKD [5-7].

In addition, two transgenic mouse lines were published. The first transgenic line expressed C148W human mutant uromodulin - which is an *UMOD* mutation known to cause UAKD in humans - under the control of the mouse *Umod* promoter in C57BL/6 inbred mice. Uromodulin accumulation was observed in TALH cells of transgenic mice, but mouse UMOD urinary excretion did not decrease and no clinical renal phenotype was observed also in aged mice [8]. The second transgenic mice expressing the corresponding C147W murine mutant uromodulin in the genetic background of FVB/N inbred mice showed the same body weight as controls. They exhibited a clinical phenotype with features of strong UAKD and renal failure with tubular necrosis at an age of 6 months [9].

In the phenotype-based Munich ENU mouse mutagenesis project using C3HeB/FeJ (C3H) inbred mice as genetic background, several mutant lines were established showing increased plasma urea levels as a parameter indicative of kidney disease [10]. Two of the lines showed dominant *Umod* mutations, i.e. lines *Umod* ^*C93F*^ and *Umod* ^*A227T*^. Similar to UAKD in humans, they exhibit a phenotype of impaired kidney function indicated by increased plasma urea and creatinine values. The functional alterations of both mutants indicated a gain-of-toxic function of mutant uromodulin, leading to TALH dysfunction due to disturbed uromodulin trafficking. Onset, severity and progression of UAKD symptoms in these mice were influenced by the type of *Umod* mutation and the allelic status. Thus, the severity of the kidney alterations was similar in *Umod* ^*C93F*^ heterozygous mutants and *Umod* ^*A227T*^ homozygous mutants. In addition, we found phenotypic alterations previously not described in mutants for this gene. Changes in body weight, body composition and bone metabolism of lines *Umod* ^*C93F*^ and *Umod* ^*A227T*^ [11,12] as well as an influence on energy metabolism in line *Umod* ^*A227T*^ [12] were revealed.

Adult homozygous mutants of both genders of line *Umod* ^*A227T*^ had a significantly lower body weight than wild-type mice. Heterozygous mutants showed an intermediate state. Analysis of body composition demonstrated that fat mass and fat content were significantly decreased in mutants whereas lean content was increased. Increased plasma calcium and alkaline phosphatase (ALP) activity were found in the mutants. To elucidate the long-term effect of hypercalciuria, analysis of the skeleton showed that bone mineral density and bone mineral content were significantly decreased in 4-month-old *Umod* ^*A227T*^ mutant animals, indicating osteopenia [12]. Analogous results with even more pronounced alterations were revealed in 9-month-old heterozygous mutants of line *Umod* ^*C93F*^ [11].

Analysis of energy metabolism in line *Umod* ^*A227T*^ at the age of 3 months revealed that body mass and body temperature were reduced in mutant mice. Metabolic rate was decreased in mutants as expected for lower body mass but food intake was significantly increased [12].

In total, we already revealed pathological effects in other organ systems than the kidneys, i.e. skeletal and metabolic alterations in *Umod* mutant mouse lines. To examine if further organ systems and/or metabolic pathways are affected by *Umod* mutations as primary or secondary effects, we describe a standardized, systemic phenotypic analysis of the two mutant mouse lines *Umod* ^*A227T*^ and *Umod* ^*C93F*^ in the German Mouse Clinic (http://www.mouseclinic.de).

## Materials and Methods

Both dominant mutant lines *Umod* ^*C93F*^ and *Umod* ^*A227T*^ were established in the Munich ENU mouse mutagenesis project using C3HeB/FeJ (C3H) inbred mice as genetic background [13]. Maintenance of the lines comprised the repeated backcross to C3H wild-type mice leading to the subsequent loss of essentially all non-causative ENU mutations that were not linked to the *Umod* mutation. The systemic, comprehensive phenotypic analysis was carried out in the German Mouse Clinic at the Helmholtz Zentrum München by using standardized examination protocols (http://www.mouseclinic.de). The analysis covers over 300 parameters in the areas of allergy, behavior, bone and cartilage, cardiovascular analysis, clinical chemistry, energy metabolism, eye analysis and vision, immunology, lung function, molecular phenotyping, neurology, nociception, pathology, and steroid metabolism. The complete protocols of the examinations are described under http://www.mouseclinic.de [14-16]. The standard workflow of the primary phenotypic analysis in the German Mouse Clinic was carried out.

First, the primary standard analysis of *Umod* ^*A227T*^ heterozygous mutant and homozygous mutant mice was carried out at an age of 2-4 months. After that, the primary standard analysis of *Umod* ^*C93F*^ heterozygous mutants was carried out at an age of 7-10 months also to especially reveal long-term secondary effects of *Umod* mutations (Table S1). For line *Umod* ^*A227T*^, 10 homozygous mutant, 10 heterozygous mutant, and 10 wild-type control littermates of each sex were used. The mice analyzed were a fifth generation backcross of the original ENU-mutated founder mouse to C3H wild-type mice. For line *Umod* ^*C93F*^, 41 heterozygous mutants and 39 wild-type control littermates were used by dividing them in two groups for two parallel phenotype analyses. The line was analyzed after backcrossing the original ENU-mutated founder mouse for more than 10 generations to C3H wild-type mice. The analysis of both mutations took place in the German Mouse Clinic at different time periods. The number of animals analyzed for both mutations was 8-11 animals per sex and genotype (except of otherwise stated in the text of the respective Results section).

Mouse husbandry was done under a continuously controlled specific pathogen-free (SPF) hygiene standard according to the FELASA recommendations [17] (http://www.felasa.eu). 

If not otherwise stated, statistical analysis of data was carried out by Student's *t*-test. Data are shown as mean ± standard error of the mean. Significant differences are indicated for *P* < 0.05, 0.01, and 0.001. In addition, the data of the feeding efficiency analysis were examined by using two-way repeated measures ANOVA.

### Ethics statement

Mouse husbandry and all tests were carried out under the approval of the responsible animal welfare authority (Regierung von Oberbayern, Germany).

## Results

Phenotypic analysis of mutant mice in the German Mouse Clinic aims at collecting and delivering with free access comprehensive phenome data of a high number of mutant mouse lines in a standardized manner. The phenotype reports of both mutant lines *Umod* ^*A227T*^ (see line “HST012”) and *Umod* ^*C93F*^ (see line “HST001”) are deposited online (http://146.107.35.38/phenomap/jsp/annotation/public/phenomap.jsf). First, the primary standard analysis of *Umod* ^*A227T*^ heterozygous mutant and homozygous mutant mice was carried out at an age of 2-4 months. As *Umod* ^*A227T*^ homozygous mutants and *Umod* ^*C93F*^ heterozygous mutants at the same age have a similar severity of the kidney alterations [11], the primary standard analysis of *Umod* ^*C93F*^ heterozygous mutants was carried out at an age of 7-10 months (Table S1).

### Additional clinical chemistry analysis

In addition to the previously published results of the clinical chemical parameters of blood plasma and urine of both *Umod* mutant lines, hematological parameters of line *Umod* ^*A227T*^ were repeatedly tested at the age of 16 and 19 weeks. Homozygous mutants and heterozygous mutants of both sexes showed small differences in hematology parameters, i.e. decreased hemoglobin, hematocrit and mean corpuscular volume at both time points versus wild-type controls (Table 1, and data not shown for the second measurement). Blood gas analysis tested at the age of 9 months indicated the absence of obvious alterations (Table 2).

**Table 1:** Hematological analysis of the lines *Umod* ^*A227T*^ and *Umod* ^C93F^.

	*Umod^A227T^*						*Umod^C93F^*			
	Males			Females			Males		Females	
Parameter	Homozygous mutants	Heterozygous mutants	Wild-type controls	Homozygous mutants	Heterozygous mutants	Wild-type controls	Heterozygous mutants	Wild-type controls	Heterozygous mutants	Wild-type controls
WBC (10³/µl)	6.7 ± 0.3	6.0 ± 0.3	6.1 ± 0.4	6.5 ± 0.4	5.8 ± 0.4	6.1 ± 0.5	6.7 ± 0.5	6.6 ± 0.2	7.1 ± 0.4	7.2 ± 0.5
RBC (10^6^/µl)	9.0 ± 0.1 ^a^	9.0 ± 0.1 ^b^	9.3 ± 0.1	8.6 ± 0.1	8.6 ± 0.1	8.6 ± 0.1	8.6 ± 0.1 ^c^	9.1 ± 0.1	9.0 ± 0.1 ^a^	9.3 ± 0.1
PLT (10³/µl)	732 ± 22 ^b^	793 ± 12	812 ± 13	780 ± 21	793 ± 12	830 ± 23	1163 ± 56	1197 ± 46	1091 ± 34	1089 ± 46
HGB (g/dl)	14.0 ± 0.1 ^c^	14.1 ± 0.1 ^c^	14.8 ± 0.1	14.1 ± 0.1	14.1 ± 0.2	14.3 ± 0.2	13.1 ± 0.1 ^c^	14.3 ± 0.1	13.7 ± 0.1 ^c^	14.9 ± 0.2
HCT (%)	46.3 ± 0.5 ^c^	46.9 ± 0.5 ^c^	49.5 ± 0.2	44.8 ± 0.6	45.2 ± 0.4	45.8 ± 0.6	44.9 ± 0.4 ^c^	49.5 ± 0.5	47.7 ± 0.4 ^c^	51.2 ± 0.6
MCV (fl)	51.5 ± 0.3 ^b^	52.1 ± 0.2 ^a^	53.0 ± 0.3	51.9 ± 0.2 ^c^	52.7 ± 0.2 ^a^	53.4 ± 0.3	52.3 ± 0.2 ^c^	54.3 ± 0.2	53.0 ± 0.1 ^c^	54.9 ± 0.2
MCH (pg)	15.5 ± 0.1	15.7 ± 0.1	15.9 ± 0.1	16.3 ± 0.1 ^a^	16.5 ± 0.1	16.7 ± 0.1	15.3 ± 0.1 ^c^	15.7 ± 0.1	15.2 ± 0.1 ^b^	15.9 ± 0.1
MCHC (g/dl)	30.2 ± 0.1	30.2 ± 0.2	29.9 ± 0.2	31.5 ± 0.2	31.3 ± 0.1	31.3 ± 0.1	29.2 ± 0.1	29.0 ± 0.1	28.8 ± 0.2	29.1 ± 0.2

WBC, white blood cell count; RBC, red blood cell count; PLT, platelet count; HGB, hemoglobin; HCT, hematocrit; MCV, mean corpuscular volume; MCH, mean corpuscular hemoglobin; MCHC, mean corpuscular hemoglobin concentration.

4-month-old mice of line *Umod* ^*A227T*^ and 8-month-old mice of line *Umod* ^*C93F*^ were tested. No. per genotype and sex: n = 8-11. Data are presented as mean ± standard error of mean. Student’s *t*-test vs. wild-type controls: ^a^ *P* < 0.05, ^b^ *P* < 0.01, ^c^ *P* < 0.001.

**Table 2:** Blood gas analysis of line *Umod* ^*A227T*^ at the age of 9 months.

Parameter	Males			Females		
	Homozygous mutants	Heterozygous mutants	Wild-type controls	Homozygous mutants	Heterozygous mutants	Wild-type controls
pH	7.33 ± 0.01	7.34 ± 0.01	7.33 ± 0.01	7.35 ± 0.01 ^b^	7.31 ± 0.01	7.31 ± 0.01
pCO_2_ (mm Hg)	46.2 ± 1.0	45.0 ± 1.1	45.5 ± 1.8	42.6 ± 0.8 ^a^	45.1 ± 1.5	46.3 ± 1.5
pO_2_ (mm Hg)	48.8 ± 2.3	56.8 ± 5.5	63.8 ± 7.2	51.8 ± 1.9 ^a^	51.8 ± 2.2	57.2 ± 1.4
sO_2_ (%)	80.1 ± 2.2 ^a^	84.1 ± 2.8	87.4 ± 1.9	84.0 ± 1.7	82.3 ± 2.1	81.4 ± 5.0
HCO_3_^-^ (mmol/l)	23.9 ± 0.6	23.5 ± 0.6	22.3 ± 1.5	23.0 ± 0.5	22.2 ± 0.6	22.5 ± 0.7
ABE (mmol/l)	-1.9 ± 0.7	-2.1 ± 0.6	-2.4 ± 0.5	-2.0 ± 0.6	-3.6 ± 0.6	-3.4 ± 0.6

sO_2_, oxygen saturation; ABE, actual base excess.

No. per genotype and sex: n = 9-10. Data are presented as mean ± standard error of mean. Student’s *t*-test vs. wild-type controls: ^a^ *P* < 0.05, ^b^ *P* < 0.01.

In line *Umod* ^*C93F*^, 35- and 43-week-old mice were used. The hematological analyses reproducibly revealed a moderate microcytic and erythropenic anemia in heterozygous mutants indicated by decreased values for red blood cell count, hemoglobin, hematocrit, mean corpuscular volume and mean corpuscular hemoglobin content (Table 1, and data not shown for the second measurement). Additional hematological analyses in *Umod* ^*C93F*^ homozygous mutants, heterozygous mutants and wild-type controls at the earlier age of 12 weeks revealed the onset of the first alterations in the hematological parameters (not shown).

In addition, the intraperitoneal glucose tolerance test (IpGTT) was carried out in line *Umod* ^*C93F*^ at the age of 8 months (n=10 per genotype and sex). Both male (*P* < 0.05) and female (*P* = 0.08) heterozygous mutants showed lower fasted plasma glucose levels than the wild-type controls (median [25% level, 75% level]: 4.7 [4.3, 5.3] mmol/l vs. 5.8 [5.2, 6.5] mmol/l in males, and 3.1 [2.9, 4.1] mmol/l vs. 3.9 [3.8, 4.8] mmol/l in females). A tendency towards a smaller area under the curve of the IpGTT was suggested in the heterozygous mutants. The results of the IpGTT were expected for the lean heterozygous mutant mice versus wild-type controls with normal body fat content. Urine analysis at the age of 9 months (n = 8-10 per genotype and sex) may indicate an increased urinary glucose excretion per gram body weight in the heterozygous mutants versus controls (excretion/24 h/25 g body weight (mean ± SD): 325 ± 131 µg vs. 227 ± 106 µg in males (*P* = 0.133), and 369 ± 64 µg vs. 283 ± 61 µg in females (*P* = 0.009)). No significant difference of urinary glucose excretion relative to the body weight was observed in 13-16 weeks-old *Umod* ^*A227T*^ homozygous mutants and heterozygous mutants compared to wild-type controls [12].

### Additional analysis of dysmorphology

In addition to the previously published differences in body weight and body composition as well as bone metabolism, systemic morphological investigation via visual inspection and X-ray analysis according to standardized protocols were carried out in both lines. In line *Umod* ^*A227T*^, visual inspection at the age of 10-12 weeks and X-ray analysis of 4-month-old animals revealed no genotype-specific differences between homozygous mutants, heterozygous mutants and wild-type controls. The same was true for the hearing ability in 2-month-old mice examined by the clickbox test using a sound of 20 kHz. Analogous results were observed in 31-36 week-old mice of line *Umod* ^*C93F*^.

### Additional analysis of energy metabolism

The alterations of the energy metabolism in 3-month-old mutants of line *Umod* ^*A227T*^ have been already published [12]. The respective plasma lipid (cholesterol, triglycerides) values were decreased in repeated analyses of homozygous mutant and heterozygous mutant mice of line *Umod* ^*A227T*^ (at 16 and 19 weeks of age) and of heterozygous mutant mice of line *Umod* ^*C93F*^ (at 35 and 43 weeks of age) compared to wild-type controls [11,12]. In contrast, in line *Umod* ^*C93F*^ at 7 weeks of age and 12 weeks of age (except of triglycerides in males), homozygous mutants with already decreased body weight showed higher plasma lipid (cholesterol, triglycerides) values compared to heterozygous mutants and/or wild-type controls. *Umod* ^*C93F*^ heterozygous mutants and wild-type controls at 84-99 weeks of age showed inconsistent results for both parameters (data not shown).

In line *Umod* ^*C93F*^, two independent analyses were carried out with two different groups of mice (n=5-7 per genotype and sex) at different time points. First, 37-week-old mice were examined both during ad libitum feeding and under food restriction conditions with the feeding efficiency protocol that was used earlier in the German Mouse Clinic. Secondly, indirect calorimetry as the common screen of energy metabolism in the German Mouse Clinic was done on 33-week-old mice under ad libitum conditions.

In the indirect calorimetry analysis (Table 3), heterozygous mutants showed reduced body mass as well as a clear reduction in body temperature by 0.9°C in males and by 0.7°C in females (*P* = 0.55). Food intake (not corrected for spillage) during 21 hours of gas exchange measurement was higher in heterozygous mutants. Both mean and minimum oxygen consumption (VO_2_) were reduced in heterozygous mutant mice. As VO_2_ mainly depends on body surface, size or mass, a linear model was calculated including body mass as independent factor. According to the model, both male and female heterozygous mutants showed a reduction in oxygen consumption by about 11% when adjusted for body mass (not shown). The mean respiratory quotient remained unaltered in heterozygous mutants and wild-type controls.

**Table 3:** Analysis of energy metabolism in line *Umod* ^C93F^.

Test	Parameter	Heterozygous mutant males	Control males	Heterozygous mutant females	Control females
Indirect calorimetry	Body weight (g)	28.9 ± 0.5 ^c^	37.2 ± 0.9	25.3 ± 0.6 ^c^	37.0 ± 1.4
	Rectal body temperature (°C)	35.3 ± 0.2 ^b^	36.2 ± 0.1	36.2 ± 0.3	36.9 ± 0.2
	Food intake (g/day)	7.6 ± 0.9	5.8 ± 0.2	7.9 ± 0.6 ^b^	5.5 ± 0.2
	Mean O_2_ consumption (ml/h)	83.4 ± 2.5 ^c^	105.9 ± 1.5	85.8 ± 2.4 ^c^	111.5 ± 2.2
	Mean respiratory quotient	0.88 ± 0.01	0.90 ± 0.01	0.92 ± 0.01 ^b^	0.88 ± 0.01
Feeding efficiency protocol,					
ad libitum	Body weight (g)	30.0 ± 0.6 ^c^	37.5 ± 1.0	26.5 ± 0.2 ^b^	36.5 ± 2.0
fasting	Body weight (g)	22.3 ± 1.1 ^c^	31.3 ± 1.0	18.9 ± 0.5 ^b^	30.5 ± 2.1
ad libitum	Rectal body temperature (°C)	35.9 ± 0.1 ^c^	36.5 ± 0.1	36.1 ± 0.2 ^b^	37.0 ± 0.1
fasting	Rectal body temperature (°C)	33.8 ± 0.7 ^a^	36.0 ± 0.1	33.6 ± 0.5 ^c^	36.5 ± 0.1
ad libitum	Food intake (g/day)	3.4 ± 0.1	3.4 ± 0.2	3.0 ± 0.1	3.3 ± 0.2
fasting	Food intake (g/day)	60% of ad lib.	60% of ad lib.	60% of ad lib.	60% of ad lib.
ad libitum	Energy content of feces (kJ/g)	16.18 ± 0.04	16.21 ± 0.06	16.13 ± 0.13	16.07 ± 0.06
fasting	Energy content of feces (kJ/g)	16.51 ± 0.08	16.64 ± 0.08	16.42 ± 0.19	16.56 ± 0.04
ad libitum	Metabolized energy (kJ/day)	50.6 ± 2.2	50.6 ± 2.4	44.2 ± 1.7	49.2 ± 2.9
fasting	Metabolized energy (kJ/day)	32.0 ± 1.6	29.9 ± 1.5	27.3 ± 0.9	29.4 ± 1.6
ad libitum	Assimilation coefficient (%)	81.7 ± 0.5	81.5 ± 0.4	81.1 ± 0.6	80.2 ± 0.5
fasting	Assimilation coefficient (%)	86.0 ± 1.1 ^b^	80.2 ± 0.3	83.4 ± 0.6 ^b^	79.9 ± 0.6

Two independent analyses (indirect calorimetry as standard screen in the German Mouse Clinic at 33 weeks of age; feeding efficiency protocol at 37 weeks of age both under ad libitum (for 7 days) and fasting (for 7 days with 60% of ad libitum consumption) conditions) were carried out with two independent groups of mice (n = 5-7 per genotype and sex). Data are presented as mean ± standard error of mean. Student’s *t*-test vs. wild-type controls: ^a^ *P* < 0.05, ^b^ *P* < 0.01, ^c^ *P* < 0.001.

Having used the feeding efficiency protocol with 5-7 mice per genotype and sex (Table 3), the data were examined by using two-way repeated measures ANOVA to analyze interactions between the two factors of genotype and feeding on the parameters of body weight (*P* = 0.076 in males, and *P* < 0.01 in females), rectal body temperature (*P* = 0.051 in males, and *P* < 0.01 in females), energy content of feces (*P* > 0.05 in males and in females), metabolized energy (*P* > 0.05 in males and in females), and assimilation coefficient (*P* < 0.01 in males, and *P* < 0.05 in females).

Heterozygous mutants also showed reduced body mass and body temperature both under ad libitum (for 7 days) and fasting (for 7 days with 60% of ad libitum consumption) conditions. During fasting no obvious difference in body mass loss between heterozygous mutant and control mice was detected compared to the ad libitum period. However, reduction in body temperature was about 0.5°C in wild-type controls compared to the ad libitum period, but much stronger in heterozygous mutants which entered states of hypothermia with a body temperature being 2-3°C below that of the controls. Another significant difference during fasting was revealed regarding energy assimilation efficiency which increased in heterozygous mutants compared to the wild-type controls. During fasting, energy content of feces increased in both genotypes compared to the respective values of the ad libitum period which is usually observed in this test (Table 3).

### Cardiovascular analysis

Non-invasive blood pressure analysis in conscious mice was carried out to determine pulse, systolic and diastolic blood pressure as well as mean arterial pressure (MAP) (Table 4). In addition, plasma concentration of the N-terminal fragment of the pro-atrial natriuretic peptide (Nt-proANP) was analyzed using the ELISA technique (Biomedica Medizinprodukte, Austria). ANP is a cardiac hormone predominantly secreted by atrial myocytes in response to cardiac filling pressures.

**Table 4:** Cardiovascular analysis of the lines *Umod* ^*A227T*^ and *Umod* ^C93F^.

	*Umod^A227T^*						*Umod^C93F^*			
	Males			Females			Males		Females	
Parameter	Homozygous mutants	Heterozygous mutants	Wild-type controls	Homozygous mutants	Heterozygous mutants	Wild-type controls	Heterozygous mutants	Wild-type controls	Heterozygous mutants	Wild-type controls
Systolic pressure (mm Hg)	108 ± 3	105 ± 3	104 ± 4	106 ± 3	110 ± 2	113 ± 3	108 ± 3	99 ± 4	106 ± 3	107 ± 3
Diastolic pressure (mm Hg)	97 ± 3	95 ± 3	94 ± 4	96 ± 4	102 ± 2	105 ± 3	96 ± 3	88 ± 4	95 ± 3	96 ± 3
Mean arterial pressure (mm Hg)	100 ± 3	98 ± 3	97 ± 4	99 ± 4	104 ± 2	107 ± 3	99 ± 3	92 ± 4	99 ± 3	99 ± 3
Pulse (bpm)	497 ± 17	496 ± 9	496 ± 7	538 ± 17	526 ± 15	534 ± 15	558 ± 12	574 ± 9	572 ± 12	579 ± 9
ECG: Fractional shortening (%)	nd	nd	nd	nd	nd	nd	35.0 ± 3.4	33.5 ± 2.5	38.2 ± 1.5	37.4 ± 2.2
ECG: Ejection fraction (%)	nd	nd	nd	nd	nd	nd	63.9 ± 4.8	62.0 ± 3.5	69.2 ± 1.9	67.5 ± 3.0

ECG, electrocardiography.

12-13 week-old mice of line *Umod* ^*A227T*^ and 32-36 week-old mice of line *Umod* ^*C93F*^ were tested. No. per genotype and sex: n = 8-10. Data are presented as mean ± standard error of mean. nd, not determined. Student’s *t*-test vs. wild-type controls: *P* > 0.05.

In line *Umod* ^*A227T*^, homozygous mutant, heterozygous mutant and wild-type control mice at an age of 12-15 weeks showed no genotype-specific differences in the blood pressure. The range of the means was 496-497 beats per minute (bpm) in males and 526-538 bpm in females for the pulse, 104-108 mm Hg in males and 106-113 mm Hg in females for the systolic blood pressure, 94-97 mm Hg in males and 96-105 mm Hg in females for the diastolic blood pressure, and 97-100 mm Hg in males and 99-107 mm Hg in females for the mean arterial pressure (MAP). Plasma Nt-proANP tended to be increased in homozygous mutants vs. wild-type controls (means ± SD: 2.64 ± 0.60 vs. 1.87 ± 1.03 nmol/l in males and 1.97 ± 0.35 vs. 1.54 ± 0.51 nmol/l in females (*P* < 0.05)). Heterozygous mutants showed intermediate means [12].

In line *Umod* ^*C93F*^, 32-38-week-old mice were used. 32-week-old heterozygous mutant and wild-type mice showed no genotype-specific differences in the blood pressure. The range of the means was 558-574 bpm in males and 572-579 bpm in females for the pulse, 99-108 mm Hg in males and 106-107 mm Hg in females for the systolic blood pressure, 88-96 mm Hg in males and 95-96 mm Hg in females for the diastolic blood pressure, and 92-99 mm Hg in males and 99 mm Hg in females for the mean arterial pressure (MAP). Plasma Nt-proANP of 38-week-old mice tended to be increased in heterozygous mutants vs. wild-type controls (1.21 ± 0.10 vs. 0.80 ± 0.07 nmol/l in males and 2.20 ± 0.27 vs. 1.73 ± 0.22 nmol/l in females).

In addition, echocardiography was done in 36-week-old mice of line *Umod* ^*C93F*^ to analyze the left ventricular function in contraction (left ventricle inner diameter (LVID) examined in systole and diastole) and pump capacity (fractional shortening, ejection fraction and left ventricular volume). Decreased end-systolic and end-diastolic left ventricular diameters were found in heterozygous mutants which is most likely related to the overall lower body weight of the heterozygous mutants. Comparing heterozygous mutants and wild-type controls, parameters representing the contractile function of the left ventricle, i.e. fractional shortening (35.0 ± 3.4% vs. 33.5 ± 2.5% in males, and 38.2 ± 1.5% vs. 37.4 ± 2.2% in females) and ejection fraction (63.9 ± 4.8% vs. 62.0 ± 3.5% in males, and 69.2 ± 1.9% vs. 67.5 ± 3.0% in females) were not different (Table 4).

### Immunology

Peripheral blood leukocytes were isolated and CD45^+^ viable cells were subsequently analyzed for the identification of main lineages (T cells, B cells, granulocytes, NK cells, monocytes) und subpopulations. Plasma antibody levels were determined simultaneously in the same samples with monoclonal anti-mouse antibodies conjugated to beads of different regions (Biorad, USA) (Table 5). The presence of rheumatoid factor and anti-DNA antibodies was evaluated by indirect ELISA with rabbit IgG (Sigma-Aldrich, Germany) and calf thymus DNA (Sigma-Aldrich), respectively, as antigens and AP-conjugated goat anti-mouse secondary antibody (Sigma-Aldrich).

**Table 5:** Immunology analysis of the lines *Umod* ^*A227T*^ and *Umod* ^C93F^.

	*Umod^A227T^*						*Umod^C93F^*			
	Males			Females			Males		Females	
Parameter	Homozygous mutants	Heterozygous mutants	Wild-type controls	Homozygous mutants	Heterozygous mutants	Wild-type controls	Heterozygous mutants	Wild-type controls	Heterozygous mutants	Wild-type controls
CD3^+^	31.1 ± 2.0	28.3 ± 1.7	27.0 ± 1.3	35.9 ± 2.3	36.4 ± 1.1	37.8 ± 1.2	25.5 ± 1.5	23.7 ± 1.7	32.7 ± 1.0 ^b^	27.7 ± 1.2
CD3^+^4^+^	17.6 ± 1.46	15.9 ± 1.3	15.2 ± 0.9	20.6 ± 1.5	20.5 ± 0.9	21.7 ± 0.7	14.1 ± 0.9	12.5 ± 1.1	18.0 ± 0.7 ^b^	14.0 ± 0.9
CD3^+^8^+^	11.5 ± 0.7	10.6 ± 0.6	10.2 ± 0.6	13.4 ± 0.7	13.3 ± 0.3	13.5 ± 0.4	8.8 ± 0.5	9.0 ± 0.6	11.5 ± 0.4	11.3 ± 0.4
CD11b^+^ Gr1^+^	19.1 ± 2.3	19.8 ± 2.5	22.6 ± 2.3	25.7 ± 2.1	27.4 ± 1.7	25.5 ± 1.3	38.1 ± 3.3	38.7 ± 2.5	27.5 ± 1.8 ^a^	33.3 ± 2.1
CD11b^+^ nonGra nonNK	8.6 ± 0.4	9.4 ± 0.5	9.8 ± 0.8	11.2 ± 0.4	11.5 ± 0.6	11.7 ± 0.8	2.0 ± 0.1	2.3 ± 0.2	1.8 ± 0.1 ^b^	2.3 ± 0.1
CD19^+^	34.9 ± 2.4	31.8 ± 1.6	33.9 ± 1.9	21.4 ± 0.9	21.3 ± 1.4	21.6 ± 0.9	25.1 ± 1.4	24.4 ± 0.8	28.4 ± 1.0	26.8 ± 1.7
CD5^-^NK^+^	4.9 ± 0.3 ^a^	6.2 ± 0.5	6.1 ± 0.3	7.2 ± 0.4	6.8 ± 0.5	6.5 ± 0.3	6.2 ± 0.4	7.4 ± 0.4	7.0 ± 0.3	6.9 ± 0.7
IgM	7774 ± 222	8017 ± 449	7784 ± 356	795 ± 73	875 ± 99	813 ± 100	2972 ± 403	2919 ± 707	nd	nd
IgA	13100 ± 397	12889 ± 356	13008 ± 476	1024 ± 103	961 ± 134	897 ± 73	3394 ± 417	3628 ± 726	4837 ± 623	5375 ± 576
IgG3	6815 ± 181	6694 ± 432	7113 ± 173	289 ± 61	382 ± 90	296 ± 54	4965 ± 2337	3419 ± 981	7798 ± 3453	9958 ± 2641
IgG1	23884 ± 482	23844 ± 339	23890 ± 487	228 ± 23	307 ± 48	243 ± 24	663 ± 46	767 ± 84	923 ± 157	914 ± 127
IgG2a	2954 ±194	2680 ± 227	3088 ± 122	597 ± 46 ^a^	554 ± 85	458 ± 40	2315 ± 430	nd	1670 ± 688	nd
IgG2b	5342 ± 627	4738 ± 428	4148 ± 459	1373 ± 226	1312 ± 92	1485 ± 183	1260 ± 141	1156 ± 156	1605 ± 164	1842 ± 56

Data are frequencies of main leukocyte subsets in blood (% of CD45^+^ viable leukocytes) and concentration (µg/ml) of antibodies of different isotypes in plasma.

3-month-old mice of line *Umod* ^*A227T*^ and 8-month-old mice of line *Umod* ^*C93F*^ were tested. No. per genotype and sex: n = 9-10. Data are presented as mean ± standard error of mean. nd, not determined. Student’s *t*-test vs. wild-type controls: ^a^ *P* < 0.05, ^b^ *P* < 0.01.

In line *Umod* ^*A227T*^, 14-week-old mice were tested. No obvious differences in the frequencies of main leukocyte populations between mutant mice compared to wild-type controls appeared. A statistically higher expression of CD62L within the CD8^+^ cell cluster occurred in female mutants (homozygous mutants vs. heterozygous mutants vs. controls: CD4^+^/CD62L^+^ (%): 23.8 ± 2.4 vs. 21.2 ± 2.1 vs. 17.8 ± 2.6 in males (not significant), and 18.6 ± 2.5 vs. 20.0 ± 2.4 (*P* < 0.05) vs. 12.3 ± 2.0 in females; CD8^+^/CD62L^+^ (%): 33.6 ± 1.9 vs. 33.2 ± 3.1 vs. 28.2 ± 3.0 in males (not significant), and 39.8 ± 3.6 (*P* < 0.05) vs. 39.0 ± 2.8 (*P* < 0.05) vs. 29.5 ± 3.1 in females). CD62L is expressed on naive T cells, as well as central memory T cells. Loss of CD62L (shedding) can occur during blood sample preparation due to release of NAD and ATP from lysed erythrocytes, and can vary substantially between inbred mouse strains. Analysis of blood plasma revealed very high antibody levels in all male mice, both mutants and controls, but no differences in the levels of antibodies in the mutants versus controls. Also no changes in autoantibodies (anti-DNA antibodies, rheumatoid factor) were observed (not shown).

In line *Umod* ^*C93F*^, 35-week-old mice were tested. The analysis revealed statistically significant differences in the frequencies of leukocyte populations between heterozygous mutant females compared to controls, namely a higher frequency of CD4^+^ T cells, which was associated with a lower proportion of granulocytes (CD11b^+^Gr1^+^). Furthermore, a higher proportion of CD62L expressing cells within the T cell cluster was found in heterozygous mutants of both sexes compared to wild-type controls (CD4^+^/CD62L^+^ (%): 25.2 ± 1.9 vs. 19.1 ± 1.7 in males (*P* < 0.05), and 34.6 ± 2.3 vs. 23.9 ± 2.5 in females (*P* < 0.01); CD8^+^/CD62L^+^ (%): 45.9 ± 2.9 vs. 37.0 ± 2.6 in males (*P* < 0.05), and 57.6 ± 1.6 vs. 49.1 ± 3.8 in females (not significant)). High levels of antibodies, but no differences between heterozygous mutants and controls were revealed (Table 5). Also no changes in autoantibodies (anti-DNA antibodies, rheumatoid factor) were observed (not shown).

### Lung function

Analysis of lung function was not carried out in line *Umod* ^*A227T*^. In line *Umod* ^*C93F*^, spontaneous breathing patterns during sleep, rest and activity were analyzed in six mice per genotype and sex at 38 weeks of age by whole body plethysmography (Table 6). The mean of all breathing frequencies (mean f) measured during the 40-minute examination period was calculated as a general parameter to assess whether the duration of rest and activity was similar in all groups. It did not differ between heterozygous mutants and wild-type controls. Specific tidal volumes and specific minute ventilations (sTV and sMV) during sleep, rest and activity, respectively, were calculated by relating the absolute values to the body weight of the animals. The values were comparable between heterozygous mutants and controls; during activity, female heterozygous mutants showed even higher sTV and sMV. Overall, observed differences were within the physiological ranges and of minor relevance. Therefore it was suggested that the mutation *Umod* ^*C93F*^ does not affect the respiratory system.

**Table 6:** Analysis of lung function in line *Umod* ^*C93F*^ at 38 weeks of age.

Parameter	Heterozygous mutant males	Control males	Heterozygous mutant females	Control females
Body weight (g)	28.9 ± 0.5 ^c^	37.5 ± 0.8	25.3 ± 0.7 ^c^	36.6 ± 1.6
Mean f (1/min)	320 ± 18	318 ± 20	316 ± 13	327 ± 20
Sleep f (1/min)	131 ± 8	142 ± 5	140 ± 2	139 ± 3
Rest f (1/min)	300 ± 9	306 ± 5	302 ± 4	301 ± 4
Activity f (1/min)	488 ± 5	489 ± 6	486 ± 3	496 ± 3
Sleep sTV (µl/g)	8.6 ± 0.4	7.8 ± 0.3	10.0 ± 0.2	9.4 ± 0.6
Rest sTV (µl/g)	5.6 ± 0.2	5.5 ± 0.2	7.2 ± 0.2	6.6 ± 0.3
Activity sTV (µl/g)	6.2 ± 0.1	5.5 ± 0.2	7.7 ± 0.2 ^b^	6.5 ± 0.2
Sleep sMV (ml/min/g)	1.0 ± 0.0	1.1 ± 0.1	1.3 ± 0.0	1.3 ± 0.1
Rest sMV (ml/min/g)	1.6 ± 0.1	1.6 ± 0.1	2.1 ± 0.1	1.9 ± 0.1
Activity sMV (ml/min/g)	3.0 ± 0.1	2.7 ± 0.1	3.7 ± 0.1 ^a^	3.2 ± 0.1

f, respiratory rates; mean f (1/min), the mean of all breathing frequencies (mean f) measured during the 40-minute examination period was calculated as a parameter to assess whether the duration of rest and activity was similar in all groups; sTV, specific tidal volumes and sMV, specific minute ventilations were calculated by relating the absolute values to the body weight of the animals.

No. per genotype and sex: n = 6. Data are presented as mean ± standard error of mean. Student’s *t*-test vs. wild-type controls: ^a^ *P* < 0.05, ^b^ *P* < 0.01, ^c^ *P* < 0.001.

### Neurology

Analysis of the basic neurological functions was not carried out in line *Umod* ^*A227T*^. In line *Umod* ^*C93F*^, 30-week-old mice were tested by modified SHIRPA, grip strength and rotarod analysis. The modified SHIRPA protocol is a semi-quantitative screening method for the overall qualitative analysis of abnormal phenotypes in mice and includes 23 test parameters each contributing to the overall assessment of general health, posture and movement, autonomic reflexes, as well as behavioral aspects. Observation of undisturbed behavior was done in a glass cylinder (11 cm in diameter). The mice were then transferred to an arena consisting of a clear Perspex box (42 × 26 × 18 cm) in which a Perspex sheet on the floor is marked with 15 squares. Locomotor activity was similar in heterozygous mutants and wild-type controls (no. of squares entered in the arena: 9.7 ± 1.3 vs. 9.0 ± 1.6 in males, and 8.7 ± 0.9 vs. 11.1 ± 1.8 in females). About half of the heterozygous mutants (6 of 10 males (χ^2^ test: not significant), and 5 of 10 females (χ^2^ test: *P* < 0.05)) and one male control animal showed weak jerks during the observation. In addition, heterozygous mutants showed increased tail elevation than control mice (7 of 10 vs. 0 of 10 in males (χ^2^ test: *P* < 0.01), and 9 of 10 vs. 5 of 10 in females (χ^2^ test: not significant)). All other SHIRPA parameters (body position, palpebral closure, lacrimation, defecation and urination during observation, as well as transfer arousal, gait, pelvic elevation, touch escape, positional passivity, trunk curl, limb grasping, pinna reflex, corneal reflex, startle response, contact righting, evidence of biting and vocalization in and above the arena) were without significant alterations.

Measurement of the forelimb grip strength to evaluate muscle performance was done with a grip strength meter system (Bioseb, France), and three trials were undertaken for each mouse and measurement within one minute. The analysis revealed a small reduction of strength in the heterozygous mutants that is most likely due to the lower body weight. Evaluation of motor coordination and balance in three consecutive trials on the rotarod (Bioseb, France) at an accelerating speed from 4 to 40 rpm for 300 sec with 15 min interval between each trial found heterozygous mutants performing even better. This may also be caused by the lower body weight. The usual improvement in the performance of the task over the three trials was observed in the heterozygous mutants and the wild-type males, but not in the wild-type females. In total, significant differences of the neurological screening were quite subtle and might hint towards a slightly higher excitation state in the heterozygous mutants.

## Discussion

Two-to-four month-old *Umod* ^*A227T*^ mutant mice and 7-10 month-old *Umod* ^*C93F*^ mutant mice were examined using a systemic and standardized, comprehensive phenotypic analysis. The two mouse lines *Umod* ^*C93F*^ and *Umod* ^*A227T*^ harbor different mutations within the *Umod* gene and exhibit a progressive renal phenotype with impairment of the urinary concentrating ability, strongly reduced fractional excretion of uric acid, altered divalent cation metabolism, and progressive morphological kidney alterations which are found in a similar manner in human UAKD. The clinical features of UAKD in humans are heterogeneous also within affected families harbouring the identical *UMOD* mutation, irrespective of type and site of the mutation that may be due to variations in the genetic background and/or the environment. The two lines *Umod* ^*C93F*^ and *Umod* ^*A227T*^ were independently established on the identical C3H inbred genetic background, housed under the same environmental conditions, and analyzed in standardized experiments. No line-specific additional sequence polymorphisms can be the cause for phenotypic alterations detected in both *Umod* mutant lines. Thus, they are models not only for determining the mutation-specific impact on the renal dysfunction in UAKD, but also to screen for additional primary and/or secondary effects of *Umod* mutations that may also appear in affected humans.

Genome-wide association studies in humans for alterations in kidney function [18] and blood pressure regulation [19] found *UMOD* locus variations including promoter variants leading to increased uromodulin expression.

The point mutation of line *Umod* ^*C93F*^ leads to the amino acid exchange C93F resulting in the loss of the putative disulfide bond C93-C105 (www.uniprot.org/uniprot/Q91X17). In humans, more than 70 *UMOD* mutations are known to cause UAKD. Many of them affect the amino acid cysteine. One is C106Y corresponding to murine C105. The severity of the kidney alterations and renal dysfunction was similar in *Umod* ^*C93F*^ heterozygous mutant mice and *Umod* ^*A227T*^ homozygous mutant mice. Therefore, adult 2-4 month-old *Umod* ^*A227T*^ heterozygous mutants and homozygous mutants as well as 7-10 month-old *Umod* ^*C93F*^ heterozygous mutants were analyzed. *Umod* ^*C93F*^ homozygous mutants with the strongest kidney alterations were not examined in the German Mouse Clinic. In affected humans, usually heterozygous *UMOD* mutations occur.

The most obvious morphological phenotype in the mutants of both lines is the reduced body weight that appears due to the highly reduced fat mass. Compared to the respective wild-type controls, the mean body weight reduction was 10% in male heterozygous mutants, 13% in female heterozygous mutants, 15% in male homozygous mutants, and 24% in female homozygous mutants of line *Umod* ^*A227T*^ at 4 months of age. In line *Umod* ^*C93F*^, the body weight reduction was 20-29% in male heterozygous mutants, and 27-35% in female heterozygous mutants at 9 months of age [11,12]. The onset of the reduced body weight of line *Umod* ^*C93F*^ was revealed at the same time as the onset of the clinical kidney alterations. Thus, at least the onset of the lower body weight may not be the consequence of chronic renal insufficiency, as young mutant mice exhibit only mild symptoms of renal dysfunction [11]. Obvious metabolic alterations were not described in the other published mouse models generated for functional studies of *Umod* which include two knockout lines [3,4] as well as two transgenic lines expressing mutant *Umod*. The transgenic line expressing C148W human mutant uromodulin exhibited no clinical renal phenotype even in aged mice [8]. The transgenic line expressing the C147W murine mutant uromodulin showed a clinical phenotype with features of UAKD and renal failure with tubular necrosis at an age of 6 months [9]. The metabolic alterations found in our both mutant mouse lines *Umod* ^*A227T*^ and *Umod* ^*C93F*^ might be due to a different expression level of uromodulin (endogenous vs. transgene expression) and/or to the different genetic background (C3HeB/FeJ versus C57BL/6 and FVB/N in the transgenic mice, respectively).

Analysis of energy metabolism in line *Umod* ^*A227T*^ at the age of 3 months under ad libitum conditions revealed that body mass and body temperature were reduced in mutant mice. Metabolic rate was decreased in mutants as expected for lower body mass but food intake was significantly increased. Metabolic fuel utilization was not different as concluded from the respiratory quotient [12]. These results were essentially confirmed in older *Umod* ^*C93F*^ heterozygous mutants by two independent analyses. It is not clear if this phenotype is the consequence of one major effect or if multiple minor effects, e.g. an increased urinary glucose excretion per gram body weight being one of them, contribute to the altered energy metabolism. Thus, the metabolic and thermoregulatory properties of both lines have to be clarified in further studies.

In addition, the pathogenic effects of the *Umod* mutations in the kidney were evaluated by comparative genome-wide transcriptome profiling using 21k cDNA microarrays (data not shown). Kidneys of *Umod* ^*A227T*^ homozygous mutants and *Umod* ^*C93F*^ heterozygous mutants (n = 4 males each) were compared to the respective wild-type controls. In line *Umod* ^*A227T*^ at the age of 17 weeks, 104 significantly regulated genes were identified. The range of the mean log_2_ ratios was 0.55 to 1.34 for the 54 up-regulated genes and -1.05 to -0.54 for the 50 down-regulated genes. Several significantly regulated genes are annotated with e.g. hemolytic anemia, glomerulosclerosis, hypertension, diabetes and tumorigenesis. In line *Umod* ^*C93F*^ at the age of 38 weeks, 54 significantly regulated genes were identified. The range of the mean log_2_ ratios was 2.57 for the single up-regulated gene *Scd1* (stearoyl-coenzyme A desaturase 1, which also represents the gene with the highest up-regulation in line *Umod* ^*A227T*^) and -2.59 to -1.36 for the 53 down-regulated genes. Several regulated genes are functionally associated with e.g. cholesterol and fatty acid metabolism, proteolysis and apoptosis as well as kidney dysfunctions. These data need to be functionally verified in further experiments. *Scd1* is expressed in various tissues and plays a key role in the general energy metabolism [20]. Further studies have to reveal the role of the increased expression of *Scd1* in the kidneys of *Umod* mutant mice.

Due to the altered kidney function and the consecutive obvious alterations of the respective blood plasma parameters found in our two *Umod* mutant lines, secondary effects in addition to the skeletal and metabolic alterations were suggested to putatively appear especially in the older *Umod* ^*C93F*^ mutants, like e.g. alterations in hematological, blood gas or blood pressure parameters, chronic inflammation of the gastrointestinal system, or uremic effects on the central nervous system.

Hematological analysis revealed a moderate microcytic and erythropenic anemia in *Umod* ^*C93F*^ mutants. Blood gas analysis as well as the cardiovascular and lung function data of *Umod* mutants at the age of 9 months showed no obvious alterations. It remains to be clarified whether the altered plasma ANP values as marker for cardiac filling pressures that appeared to be increased by tendency in *Umod* mutants of both lines may be also caused by the lower body weight. At least no functional consequences thereof were observed in the cardiovascular analyses. The behavioral (data not shown) and neurological results of both *Umod* ^*A227T*^ and *Umod* ^*C93F*^ mutants were within the physiological range of C3H mice and did not permit to define an altered behavioral pattern for mutant animals. The appearance of weak jerks during the observation in about half of the *Umod* ^*C93F*^ heterozygous mutants remains to be further analyzed.

Immunological analyses of older *Umod* mutants revealed small changes in the frequencies of leukocyte populations. Furthermore, a higher proportion of CD62L expressing cells within the T cell cluster was found that represent the naive T cell compartment, newly produced in the thymus. This is an often observed phenotype in various mutant lines. In total, no indications were observed for the appearance of major inflammatory processes in the mutant mice. Urinary UMOD excretion was similar in *Umod* ^*A227T*^ homozygous mutants and *Umod* ^*C93F*^ heterozygous mutants, and markedly decreased compared to wild-type controls [11]. Recently the role of UMOD as a regulator of renal and systemic immunity was proposed as homozygous *Umod* knockout mice showed absolutely and relatively enlarged spleens with white-pulp macrophage infiltration as well as an increase of serum cytokines via decreased glomerular clearance and loss of urinary cytokine trapping [21]. The plasma parameters measured in *Umod* ^*A227T*^ and *Umod* ^*C93F*^ mutants did not give hints to the proposed role of UMOD as a regulator of immunity in the setting of UAKD with present UMOD expression but disturbed protein maturation. In addition, spleen weights relative to the body weight or carcass weight showed no differences in *Umod* ^*A227T*^ homozygous mutants and wild-type controls at 3 months and 22 months of age (not shown). Analogously, *Umod* ^*C93F*^ heterozygous mutants with severely decreased urinary UMOD excretion and wild-type controls at 1 year of age and 84-99 weeks of age showed the same spleen weight relative to the carcass weight (1 year of age (mean ± SD): 0.73 ± 0.05% vs. 0.80 ± 0.15% in males, and 1.08 ± 0.18% vs. 1.04 ± 0.13% in females; n = 8-11 per genotype and sex).

In conclusion, uromodulin is selectively expressed in the kidney and amino acid-changing mutations of *Umod* lead to the clinical symptoms of UAKD, a slowly progressive renal disease of mild urinary concentration defect, reduced fractional excretion of uric acid and morphological tubulointerstitial kidney alterations. The systemic phenotypic analysis of the two mutant mouse lines *Umod* ^*A227T*^ and *Umod* ^*C93F*^ on the C3H inbred genetic background reproducibly revealed alterations in body weight, body composition, bone metabolism, and energy metabolism. In older mutants, a moderate microcytic and erythropenic anemia was revealed. The other analyses in 7-10 months-old mice showed single small additional effects. Thus, depending on the genetic background, long-term effects additional to the kidney lesions might also appear in affected humans.

## Supporting Information

Table S1 **Time points of the phenotypic analyses described for the lines *Umod*^*A227T*^ and *Umod*^*C93F*^ in the German Mouse Clinic (GMC).**
(DOCX)
