# Supplementary material for: Standardized, Systemic Phenotypic Analysis of Umod C93F and Umod A227T Mutant Mice
Source: PLoS One. 2013 Oct 24;8(10):e78337. doi: 10.1371/journal.pone.0078337 (PMC3813435; doi:10.1371/journal.pone.0078337)
Supplement: Table S1 — Time points of the phenotypic analyses described for the lines UmodA227T and UmodC93F in the German Mouse Clinic (GMC). (DOCX) [file pone.0078337.s001.docx]

Table S1. Time points of the phenotypic analyses described for the lines *Umod^A227T^* and *Umod^C93F^* in the German Mouse Clinic (GMC)

| Phenotypic analysis |  | Line *Umod^A227T^* | Line *Umod^C93F^* |
| --- | --- | --- | --- |
| Standard GMC analysis |  | 2-4 months | 7-10 months |
| Clinical chemistry | hematology | 16 and 19 weeks | 12, 35 and 43 weeks |
|  | IpGTT | not done | 8 months |
|  | urinary glucose | 13-16 weeks ^1^ | 9 months |
|  | blood gas analysis | 9 months | not done |
| Dysmorphology | clickbox test | 2 months | 31-36 weeks |
|  | visual inspection | 10-12 weeks | 31-36 weeks |
|  | X-ray | 4 months | 31-36 weeks |
| Energy metabolism | plasma lipids | 16 and 19 weeks ^1^ | 7 and 12 weeks,  35 and 43 weeks ^1^,  84-99 weeks |
|  | indirect calorimetry | data (3 months) previously published ^1^ | 33 weeks |
|  | feeding efficiency protocol | not done | 37 weeks |
| Cardiovascular analysis | blood pressure | 12-15 weeks | 32 weeks |
|  | echocardiography | not done | 36 weeks |
|  | Nt-proANP | 4 months ^1^ | 38 weeks |
| Immunology |  | 14 weeks | 35 weeks |
| Lung function |  | not done | 38 weeks |
| Neurology |  | not done | 30 weeks |
| Transcriptome analysis |  | 17 weeks | 38 weeks |

IpGTT, intraperitoneal glucose tolerance test; Nt-proANP, plasma concentration of the N-terminal fragment of the pro-atrial natriuretic peptide.

^1^ Cited data that was previously published [11,12].
